# The Putative SLC Transporters *Mfsd5* and *Mfsd11* Are Abundantly Expressed in the Mouse Brain and Have a Potential Role in Energy Homeostasis

**DOI:** 10.1371/journal.pone.0156912

**Published:** 2016-06-07

**Authors:** Emelie Perland, Emilia Lekholm, Mikaela M. Eriksson, Sonchita Bagchi, Vasiliki Arapi, Robert Fredriksson

**Affiliations:** 1 Unit of Functional Pharmacology, Department of Neuroscience, Uppsala University, Uppsala, Sweden; 2 Unit of Molecular Neuropharmacology, Department of Pharmaceutical Bioscience, Uppsala University, Uppsala, Sweden; Federal University of Rio de Janeiro, BRAZIL

## Abstract

**Background:**

Solute carriers (SLCs) are membrane bound transporters responsible for the movement of soluble molecules such as amino acids, ions, nucleotides, neurotransmitters and oligopeptides over cellular membranes. At present, there are 395 SLCs identified in humans, where about 40% are still uncharacterized with unknown expression and/or function(s). Here we have studied two uncharacterized atypical SLCs that belong to the Major Facilitator Superfamily Pfam clan, Major facilitator superfamily domain 5 (MFSD5) and Major facilitator superfamily domain 11 (MFSD11). We provide fundamental information about the histology in mice as well as data supporting their disposition to regulate expression levels to keep the energy homeostasis.

**Results:**

In mice subjected to starvation or high-fat diet, the mRNA expression of *Mfsd5* was significantly down-regulated (P<0.001) in food regulatory brain areas whereas *Mfsd11* was significantly up-regulated in mice subjected to either starvation (P<0.01) or high-fat diet (P<0.001). qRT-PCR analysis on wild type tissues demonstrated that both *Mfsd5* and *Mfsd11* have a wide central and peripheral mRNA distribution, and immunohistochemistry was utilized to display the abundant protein expression in the mouse embryo and the adult mouse brain. Both proteins are expressed in excitatory and inhibitory neurons, but not in astrocytes.

**Conclusions:**

*Mfsd5* and *Mfsd11* are both affected by altered energy homeostasis, suggesting plausible involvement in the energy regulation. Moreover, the first histological mapping of MFSD5 and MFSD11 shows ubiquitous expression in the periphery and the central nervous system of mice, where the proteins are expressed in excitatory and inhibitory mouse brain neurons.

## Introduction

Among all proteins in the human genome around 27% are membrane bound [1], and within this group the solute carriers (SLCs) are the second largest family consisting of at least 395 members in humans [2]. Currently, the SLC superfamily is divided into 52 subfamilies [2], where members belonging to a specific subfamily share 20–25% sequence homology [3]. By phylogenetic analyses 15 human SLC families are further clustered into four main groups, designated α-, β-, γ- and δ [4]. The α-group is the largest with seven SLC families (SLC2, 16, 17, 18, 22, 37 and 46), together with the synaptic vesicle 2 (SV2) proteins. The β-group includes three amino acid transporter families (SL32, 36 and 38), while the γ- (SLC7 and 12) and δ-group (SLC 8 and 24) include two families each.

The majority of the mammalian SLC proteins can also be classified into three Pfam clans based on sequence homology; the major facilitator superfamily (MFS), amino acid- polyamine organocation (APC) and the monovalent cation:proton antiporter (CPA)/anion transporter (AT) clan [5]. The MFS clan is one of the largest groups of phylogenetically related membrane proteins [6] and the largest group of phylogenetically related SLCs in humans [5]. It is also one of the most functionally diverse superfamilies among the transporter proteins [6,7]. Human MFS proteins are closely related to SLCs based on sequence similarity measures [4] and some of these are referred to as atypical SLCs [8]. Many atypical SLCs do not have a SLC name or symbol, but rather a name by the MFSD nomenclature or a name from a large scale sequencing project [4].

The SLC family contains a variety of transporter proteins with distinct expressions patterns in cytoplasmic- and organelle membranes [3,8]. They also translocate a broad range of substrates, including amino acids, ions, nucleotides, neurotransmitters or oligopeptides [2,6]. Within the SLC- and MFS family, transporters can function as uniporters, symporters or antiporters [3,7,9], and most members have 12 putative transmembrane α-helical domains [4,9–11] to carry out this transport. Notably is that many SLCs are evolutionary conserved with homologues in prokaryotes, invertebrates and mammals [5,8], suggesting that they are, or have been, important for survival during evolution. Despite their important role, about 40% of all known SLCs are still orphan transporters [4] with unknown cellular location and/or substrate profiles.

In this article we focus on two novel genes, *Major facilitator superfamily domain 5* (*Mfsd5*) and *Major facilitator superfamily domain 11* (*Mfsd11*). Both proteins are putative SLC permeases belonging to the MFS clan and their cellular localization and transported substrate(s) are yet not elucidated. MFSD5 is an atypical SLC, phylogenetically belonging to the α-group [8], whereas MFSD11 is not classified into a group. Both proteins are thought to have 12 transmembrane domains based on protein prediction [12]. Previous quantitative real time PCR (qRT-PCR) analysis of *Mfsd5* in adult rats [8] and *in situ* hybridization in mouse embryos [13] demonstrated that it is ubiquitously expressed. Overexpression studies using human *MFSD5* (called HsMOT2) in *Saccharomyces* cells, demonstrated that it function as an ion transporter, with molybdate ions as a main substrate [14]. Molybdate (MoO_4_^2-^) is the bioavailable form of molybdenum and plays a role in the active site of more than 50 pterin-containing enzymes [15,16]. Disturbances in enzyme activity could be the reason why male homozygous *Mfsd5* knockout mice have more anxious or depressed behaviour compared to littermate controls [13].

MFSD5 is also reported to interact with the incretin hormone glucagon-like peptide 1 receptor (GLP-1R) in a cell-based study where GLP-1R was overexpressed in a CHO cell line [17]. MFSD5 is thereby one of several potential manipulators of maintaining the glucose homeostasis and pancreatic β-cell proliferation. Interestingly, when mouse hypothalamic N25 cells are starved on all amino acids, *Mfsd11* gene expression is up-regulated as seen using Genechip^®^ ST microarray and qRT-PCR [18]. Hence there are previous data indicating that both MFSD5 and MFSD11 are involved in altered energy homeostasis.

Here we utilized qRT-PCR to show that the expression levels of both *Mfsd5* and *Mfsd11* are changed in mice subjected to starvation or high-fat diet (HFD) in specific brain areas and larger brain sections. Overall, the mRNA levels for *Mfsd5* were significantly down-regulated by both starvation and HFD in brain sections containing areas involved in food intake and reward, while *Mfsd11* expression was up-regulated by starvation and HFD in brain sections. Specifically, we showed changed expression of *Mfsd5* in cortex and hypothalamus from food restricted mice, while no alterations were measured for *Mfsd11*.

Based on a phylogenetic analysis, MFSD11 was closely related to MFSD5 in humans, as well as to SLC families having organic substrate profiles. We also provide a detailed histological characterization of *Mfsd5* and *Mfsd11* at the mRNA and protein level in the mouse brain as well as in peripheral organs. MFSD5 and MFSD11 staining were seen in abundance during embryogenesis and in adults, and they were expressed in both excitatory and inhibitory neurons.

## Material and Methods

### Animals

All animal procedures in this study were approved by the local ethical committee in Uppsala (Uppsala Djurförsöksetiska Nämnd, Uppsala district court) (Permit Number C419/12 and C67/13), and conformed to the guidelines of European Communities Council Directive (2010/63). All procedures involving mice were performed in such a way as to minimize suffering. Adult male mice were used for all trials. All animals were maintained in a temperature-controlled room on a 12h light-dark cycle where they had free access to food and water at all times unless anything else is stated. C57BL/6J mice (Taconic M&B, Denmark) were used as wild type (wt) in all trials. Additionally, heterozygous Tg(SLC32a1-EGFP)EM128Gsat/Mmucd mice (VIAAT mice) (sperm from MMRRC, injected at Portalen Nord, Karolinska institutet) were used for primary cultures. In these mice the VIAAT protein [19] promoter drives the expression of eGFP, hence the inhibitory neurons are pre-labelled.

### Tissue dissection from mice in diet studies

Adult male mice were sacrificed by cervical dislocation during the light period. All dissections were performed on ice and tissues were collected within 10min after animal euthanasia. Three groups were studied; 1) control mice that were fed standard chow (R3, Lantmännen, Sweden) until dissection, 2) starved mice that were fed standard chow, but were food deprived for 24h before dissection and 3) mice that were fed high fat western diet (HFD) (R638, Lantmännen, Sweden), during eight weeks prior dissection to induce obesity The weights of the HFD mice were monitored weakly and compared with normal chow fed mice. One-way ANOVA was calculated to ensure that the obese mice were significantly heavier than the controls at the day of dissection, significance level was set at P<0.05. All mice had access to water at all time. For specific brain areas: n = 4 per group. For the brain sections: controls n = 6, starved mice n = 4 and HFD mice n = 6. The brain sections were cut using a brain matrix (Alto, 1mm). Tissues were placed in RNA-later (Invitrogen) for 2h at RT before frozen at -80°C.

### Tissue and blood preparation for RNA extraction from wt mice

Adult wt male mice raised on standard chow were also used to isolate central- and peripheral tissues (n = 5 per organ), while females were utilized for female genitalia (n = 5). Mice were sacrificed by cervical dislocation and all dissections were performed on ice before tissues were placed in RNA-later (Qiagen) for 2h at RT and frozen at -80°C until further processed. For blood sampling, mice were sacrificed by cervical dislocation before a cardiac puncture was performed. The blood was mixed with EDTA (1.5mg/ml blood, VWR) before centrifuged in a Heraesus Fresco 21 centrifuge for 10min, 4°C, at maximum speed, 21100x g; the pellet was used for RNA extraction.

### RNA extraction

RNA was extracted from individual samples by using Absolutely RNA Miniprep Kit (Agilent Technologies). Briefly, the tissues were mixed with 1mm RNAse free glass beads, Lysis Buffer and β-mercaptoethanol and homogenized using a Bullet blender (Next advance, USA). The homogenate was spun through a pre-filter spin cup in a Heraesus Fresco 21 centrifuge at maximum speed at RT, before mixed with 70% ethanol in a 1:1 ratio (Solveco) for RNA precipitation. The solution was centrifuged through a RNA binding spin cup. The filter was washed in salt buffers and RNase-Free DNase 1 and allowed to incubate for 15min at 37°C. Additional salt buffer washes were performed prior to RNA elution. Concentration was measured using a ND-1000 spectrophotometer (NanoDrop Technologies).

### First-strand cDNA synthesis

Extracted RNA was used as template for two-step qRT-PCR reactions using the 2xRT Reaction mix and RT enzyme mix (SuperScript III first-strand synthesis supermix, Invitrogen). They were mixed, to which 1 μg RNA template was added and the final volume was adjusted to 20 μL with DEPC-treated water. Samples were incubated for 10min at 25°C followed by 30min at 50°C and the reaction was terminated by 5min incubation at 85°C before cooled on ice. 2U E.coli RNase H was added to each reaction before additional 20min incubation at 37°C. cDNA from the same section/region, but from different animals, were pooled and diluted to 5 ng/μl RNA in sterile water.

### Primer design and quantitative real-time PCR

All primers were designed using Beacon Design 8 (Premier Biosoft, Palo Alto, CA, USA). For sample amplification the primers used were *Mfsd5* forward 5’-tgttgggtgtcatacaagc-3’ and reverse 5’-ggtctagcacaggtgtcc-3’ and *Mfsd11* forward 5’-tgtggagtatgcctcact-3’ and reverse 5’-ttcagaaagtcattcccaga-3’. Six different reference genes were used: *glyceraldehyde-3-phosphate dehydrogenase* (*Gapdh*) forward 5’-gccttccgtgttcctacc-3’, reverse 5’-gcctgcttcaccaccttc-3’, *beta tubulin* 4B (*bTub*) forward 5’-agtgctcctcttctacag-3’, reverse 5’-tatctccgtggtaagtgc-3’, *ribosomal protein L19* (*Rpl19*) forward 5’-aatcgccaatgccaactc-3’, reverse 5’-ggaatggacagtcacagg-3’, *histone cluster 1* (*H3a)* forward 5’-ccttgtgggtctgtttga-3’, reverse 5’-cagttggatgtccttggg-3’, *peptidylpropyl isomeras A* (*Cyclo*) forward 5’-tttgggaaggtgaaagaagg-3’, reverse 5’-acagaaggaatggtttgatgg-3’ and *actin-related protein 1B* (*Actb*) forward 5’-ccttcttgggtatggaatcctgtg-3’, reverse 5’-cagcactgtgttggcatagagg-3’.

Final volume for each qRT-PCR reaction was 20 μl; 1 μl pooled cDNA (5ng/μl), 0.05 μl of each primer (100pmol/μl), 2 μl 10x reaction buffer (Biotools, Techtum), 0.2 μl of 25mM dNTP mix (Fermentas), 1.6 μl 50mM MgCl_2_, 1μl DMSO, 0.5 μl of SYBR Green (1:50000; Invitrogen) in TE buffer (pH 7.8) and 0.08 μl of Taq polymerase (Biotools, Techtum). iCycler real-time detection instrument (Bio-Rad Laboratories) was used and the reaction followed these conditions: initial denaturation for 30 sec at 94°C followed by 50 cycles of 10 sec at 94°C, 30 sec at 55–61°C (optimal temperature for each primer pair) and 30 sec at 72°C. Thereafter a melting curve was performed by 81 cycles of 10 sec intervals where the temperature increased 0.5°C per cycle, starting from 55°C. All qRT-PCR reactions were performed in triplicates, in addition negative and positive controls were included on each plate. All experiments were repeated twice.

### Analysis of qRT-PCR data from mice subjected to different diets

MyIQ (Bio-Rad Laboratories) software was used for obtaining CT-values for all samples. Primer efficiency was calculated for each run using LinRegPCR software, followed by Grubbs test (GraphPad software) to remove outliers in the efficiency calculations, before correcting the samples for primer efficiency. The GeNorm protocol [20] was then used to calculate the geometric mean of the three housekeeping genes, *Gapdh*, *H3a* and *Actb*, as subsequently was used as normalisation factors for each tissue. The CT-values for *Mfsd5* and *Mfsd11* were normalized and plotted (±SD). Differences in gene expression between the diets were analysed with one-way ANOVA, where Bonferroni’s multiple comparison test was used for post-hoc analysis. The chow fed mice were considered as normal and P>0.05 was used as significance. Statistical analyses and enclosed graphs have been done using software GraphPad Prism 5.

### Analysis of wt mouse qRT-PCR panel

Followed the same procedure as previously; after primer efficiency calculations, the normalization factors were calculated in GeNorm using the following reference genes: *Gapdh*, *bTub*, *Rpl19*, *Cyclo* and *Actb*. The normalized mRNA expression (±SD) was plotted.

### Phylogenetic analysis

All human SLC sequences of MFS type (i.e. family 2, 15, 16, 17, 18, 22, 29, 37, 40, 43, 45, 46) obtained according to the SLC tables database [21] were downloaded and combined with human *SV2A*, *SV2B*, *SV2C*, *SVOP*, *SVOPL*, *MFSD5* and *MFSD11* sequences into a multiple sequence alignment using tcoffee [22]. From this alignment a Sequence Hidden Markov Model (sHMM) was calculated with HMMbuild from the HMMER package [23], which was calibrated using the hmmcalibrate, and finally searched against the protein datasets listed in Table 1 using the hmmscan.pl script (http://code.google.com/p/interproscan/source/browse/trunk/core/jms-implementation/support-mini-x86-32/bin/superfamily/1.75/hmmscan.pl?r=1397). All hits from each species were combined with the human dataset and analysed using RAxML [24] with default settings. From these phylogenetic trees, all *MFSD5* and *MFSD11* orthologous were identified, and these orthologous were subsequently combined with the entire human dataset in a multiple sequence alignment using tcoffee. The phylogenetic relationships between these sequences were inferred with RAxML [24] using default settings to obtain a tree.

**Table 1:** Description of the genomes used for MFSD5 and MFSD11 phylogenetic analysis.

Species	Common name	Data version
*A*. *carolinensis*	Green anole	2.0.61.pep.all
*C*. *elegans*	Roundworm	WS220.61.pep.all
*C*. *intestinalis*	Sea squirt/Vase tunicate	JGI2.61.pep.all
*C*. *savignyi*	Pasific transparent sea squirt	CSAV2.0.61
*D*. *rerio*	Zebrafish	Zv9.61.pep.al
*D*. *melanogaster*	Fruit fly	BDGP5.73.pep.all
*G*. *gallus*	Chicken	WASHUC2.61.pep.all
*G*. *aculeatus*	Three-spined stickleback	BROADS1.61.pep.all
*Homo sapiens*	Human	GRCh37.73.pep.all
*M*. *musculus*	Mouse	NCBIM37.61.pep.all
*T*. *nigroviridis*	Green spotted puffer	TETRAODON8.61.pep.al

All genomes were obtained from Ensemble [25] except the *Nematostella vectensis* which was gathered from JGI genome portal [26].

### Tissue preparation for Western Blot

Approximately 0.25mg tissue from whole mouse brain or kidney was used per sample. The tissue was homogenized by adding glass beads and homogenization buffer (50mM Tris, 150mM NaCL, 4mM MgCl, 0.5mM EDTA, 2% Triton-X, 1mM Protease inhibitor PMSF (Roche)) and run for 3min in a Bullet blender. After a quick spin, the supernatant was transferred to new tubes and spun at 14000x g for 15min at 4°C. The supernatant was stored at -20°C.

### Western blot

20μg (for MFSD5) and 100μg (for MFSD11) from brain and kidney protein samples were mixed with Laemmlis Sample Buffer (Bio-Rad) and various concentrations of 2-mercaptoethanol (Fluka), and loaded onto 12% TGX Miniprotean gels (Bio-Rad) and allowed to run at 100V. A pre-stained molecular weight marker was used as reference (Thermo Fischer). The transfer was performed either by blotting onto PVDF membrane (Immobilon-P, Millipore) at 4°C at 50V for 60min or by using the Trans-Blot^®^ Turbo^™^ Mini PVDF Transfer Packs and Trans-blot Turbo Transfer system (Bio-Rad), following the manufactures instructions. The membrane was then incubated in 5% milk (Blotting grade blocker, Bio-Rad) TTBS blocking solution for 60min. Western blot was run with the polyclonal primary antibodies anti-MFSD5 (1:500, goat, Santa Cruz, sc-243473) and anti-MFSD11 (1:100, rabbit, Sigma-Aldrich, HPA022001), as these antibodies were utilized for subsequent staining. The antibodies were added to the membrane and allowed to bind over night at 4°C. After 3x10min wash with TTBS the membrane was incubated at RT for 60min with HRP coupled secondary antibodies (anti-goat and anti-rabbit (Invitrogen), diluted 1:10000 in blocking solution). The membrane was developed using Clarity Western ECL Substrate (Bio-Rad) and visualized using a CCD camera (Bio-Rad), staining was compared to the molecular weight marker.

### Tissue collection from adult mice and e14-15 embryos for immunohistochemistry

Adult mice received an intraperitoneal injection with 0.5mg/g body weight Sodium Pentobarbital (Apoteket Farmaci, Sweden) before transcardial perfusion was performed by pumping phosphate-buffered saline (PBS) through the left chamber of the heart followed by 4% formaldehyde (Histolab, Sweden) for fixation. The brains were excised and stored in 4% formaldehyde overnight before either floating sectioned or paraffin embedded followed by microtome sectioning. For embryo collection, pregnant females were sacrificed by an intraperitoneal injection of 0.5mg/g body weight Sodium Pentobarbital at e14-15. The embryos were removed from the uterus and kept in cold HBSS (Gibco) during separation from the yolk sac and placenta, before placed in 4% formaldehyde for 2h at ice followed by paraffin embedding.

### Tissue preparation and sectioning

Brains and e14-15 embryos were embedded in paraffin by a Tissue Tek Vacuum Infiltration Process. Samples were post-fixated in 10% zinc formalin for 40min (brains) or 6h (embryos) at 40°C, dehydrated in 70% ethanol (Solveco) for 1h at 40°C followed by several isopropanol (Solveco) baths at 40°C before embedded in paraffin (DaLab, Sweden). 7μm coronal brain- and sagittal embryo sections were then cut in a HM355S microtome (Thermo Scientific), then dried over night at 37°C and finally stored at 4°C. For floating sections, fixed brains were rinsed in PBS before mounted in 4% agarose (Conda). 70μm coronal sections were cut in a vibratome (Leica Microsystems) and stored in Tris-buffered saline (TBS) until stained.

### Fluorescent double immunohistochemistry on paraffin brain- and embryo sections

Brain and e14-15 embryo sections were deparaffinised in X-tra Solve (Medite, Dalab) followed by rehydration through an ethanol series (100, 95, 75, 50, 25% and water), and an antigen retrieval procedure by boiling them for 10min in 0.01mM citric acid (Sigma-Aldrich), pH 6.0. When cooled down, sections were placed in a humidity chamber, washed in PBS and incubated with primary antibodies diluted in supermix (TBS, 0.25% gelatine, 0.5% Triton X-100) overnight at 4°C. Anti-MFSD5 (1:400, Santa Cruz) and anti-MFSD11 (1:80, Sigma-Aldrich) were continually used for all stainings. These were co-stained with anti-MFSD5 (1:50, rabbit, MyBioSource, MBS9202695, polyclonal) and anti-MFSD11 (1:80, goat, Santa Cruz, sc-243472, polyclonal) to ensure properly working antibodies in tissues before additional double immunostainings. The following cellular markers were also utilized: monoclonal anti-NeuN (1:400, mouse, Millipore, MAB377), anti-MAP2 (1:500, mouse, Sigma-Aldrich, M4403) and anti-GFAP (1:800, mouse, Millipore MAB360). Sections were then washed in PBS before incubated in secondary antibodies (Alexa 488 donkey-anti-goat, Alexa 488 goat-anti-rabbit and Alexa 594 donkey-anti-mouse (Invitrogen)) diluted 1:800 in supermix for 2h at RT. Negative controls without primary antibodies were also run for all secondary antibodies. The sections were further washed in PBS, followed by DAPI (Sigma-Aldrich) staining, diluted 1:1250 in PBS, for 10min prior mounting in mowiol anti-fade media (25g mowiol 4–88 in 100mL 1xPBS, pH 8.0, 50mL glycerol, 3mL of 1% Thimerosal, and 100μg/ml *n*-propyl gallate in (all from Sigma-Aldrich)). An Olympus microscope BX53 with an Olympus DP73 camera was used for the antibody verification. The micrographs were acquired by cellSens Dimension software. For the cellular marker staining, a fluorescent microscope (Zeiss Axioplan2 imaging) connected to a camera (AxioCam HRm) with the Carl Zeiss AxioVision version 4.7software was used. Embryos were scanned in a Mirax pannoramic scanner (3dHistech) with the pannoramic software.

### Proximity Ligation Assay

Proximity ligation assay (PLA) was run on paraffin embedded mouse brain section as previously described **[27]**. The following antibody parameters were used: Anti-MFSD5 (1:400, Santa Cruz) vs anti-MFSD5 (1:50, MyBioSource) and anti-MFSD11 (1:80, Sigma-Aldrich) vs anti-MFSD11 (1:80, Santa Cruz). DAPI was included in all set ups. Images were taken using the Zeiss Axioplan2 fluorescent microscope.

### DAB immunohistochemistry on floating sections

Floating sections were washed in Tris-buffered saline (TBS) followed by incubation in 10% Methanol (Sigma-Aldrich) and 4% hydrogen peroxide (Merck, Germany) diluted in TBS for 10min. After additional washes tissues were blocked for 1h in supermix before the primary antibodies (anti-MFSD5 (1:500, Santa Cruz), anti-MFSD11 (1:500, Sigma-Aldrich)), diluted in supermix were added and allowed to incubate at 4°C overnight. Sections were then washed in TBS and secondary biotinylated antibodies (1:400, Vector laboratories) were added and incubated for 1h at RT. After additional TBS washes, 1h incubation in the ABC kit solution (Vector laboratories) was performed. The samples were then developed in a solution consisting of one DAB tablet (Sigma-Aldrich) dissolved in 12,5ml TBS, 3.8% NiCl and 0.03% hydrogen peroxide added directly prior development for a maximum of 10min, the reactions were stopped by TBS washes. Sections were transferred to gelatine-coated slides (Superfrost, Menzel-Gläser, Thermo Scientific) and dried overnight before dehydrated by an ethanol series completed with Xylene (Sigma-Aldrich) and mounting in DPX (Sigma-Aldrich). Sections stained without primary antibodies were used as negative controls. Samples were scanned in a Pannoramic midi scanner using bregma according to *The mouse brain* atlas [28].

### Embryo collections for primary cell culture

Wt and VIAAT male mice were mated with wt females, and at e14-15 the females were sacrificed by cervical dislocation. Embryos were removed from the uterus and kept in cold HBSS (Gibco) during separation from the yolk sac and placenta. Embryos were decapitated in 1x PBS with 10mM glucose before cortex was dissected under a Leica CLS 100 LED microscope. The cortices were chemically dissociated in 10μg/mL DNase (Invitrogen)/0.5mg/ml Papain (Sigma-Aldrich-Aldrich), diluted in PBS-10mM glucose, for 30min at 37°C, 5% CO_2_. Tissues were then rinsed in plating media (DMEM-F12 (Gibco), 10% FBS (Gibco), 2mM L-glutamine (Invitrogen), 1mM Na-Pyruvate (Invitrogen) and 1% penicillin/streptomycin (Invitrogen)) before mechanically dissociated by pipetting up and down with a Pasteur pipette and filtered through a 70μm nylon cell strainer (BD Stockholm) to remove remaining cell clusters. Cells were plated at a density of 7.5*10^4^ cells on poly-L-lysine (Sigma-Aldrich-Aldrich) coated cover slips (12mm, #1.5, Menzel-Gläser), kept in 24 multi well plates (BD) and incubated for 3h at 37°C, 5% CO_2_. Plating media was then replaced with growth media (Neurobasal-A (Gibco,), 2mM L-Glutamine, 1mM Na-Pyruvate, 1% penicillin/streptomycin and 2% B27 (Invitrogen)). Two third of the growth media were changed every third day and at the tenth day, cells were rinsed with 37°C PBS-10mM glucose before fixated in 4% formaldehyde for 10min. Cells were kept at 4°C in PBS till stained.

### Immunocytochemistry on embryonic primary cell cultures

Cells were prepared as described above, and at the tenth day the cells were rinsed with 37°C PBS-10mM glucose before fixated in 4% formaldehyde for 10min. Cells were washed in PBS and blocked in 5% milk protein (Bio-Rad) diluted in PBS for 1h at RT followed by primary antibody (diluted in 5% milk blocking) incubation with agitation at 4°C overnight. Double-staining using anti-MFSD5 (1:400, Santa Cruz) or anti-MFSD11 (1:80, Sigma-Aldrich) with monoclonal anti-glutaminase (1:100, mouse, Abcam, ab60709) was executed on wt cells to visualize the expression of MFSD5 and MFSD11 in excitatory cells. Glutaminase is an enzyme that, in its phosphate-activated state, generates glutamate and ammonia from glutamine in glutamatergic neurons. Single primary antibody staining with anti-MFSD5 (1:400, Santa Cruz) or anti-MFSD11 (1:80, Sigma-Aldrich) was performed on the VIAAT embryonic cell cultures, in which the VIAAT positive neurons were genetically pre-labelled with eGFP. VIAAT is expressed in inhibitory neurons [19] and was hence used as a marker for inhibitory cells. After primary antibody incubation the cells were washed in PBS and incubated for 2h at RT on circulation in secondary antibodies diluted 1:800 in 5% milk-blocking. Alexa 488 donkey-anti-goat, Alexa 488, goat-anti-rabbit, Alexa 594, donkey-anti-mouse (Invitrogen) were used for wt cell staining and A594 donkey-anti-goat and A594 donkey-anti-rabbit were used for the VIAAT-eGFP cultures. DAPI was then added for 10min followed by PBS rinses and mounting in mowiol anti-fade media. Pictures were taken in a Zeiss LSM 710 confocal microscope, where Zen Black was used as software.

## Results

### Changes in mRNA expression of *Mfsd5* and *Mfsd11* by food intake

**Fig 1:**
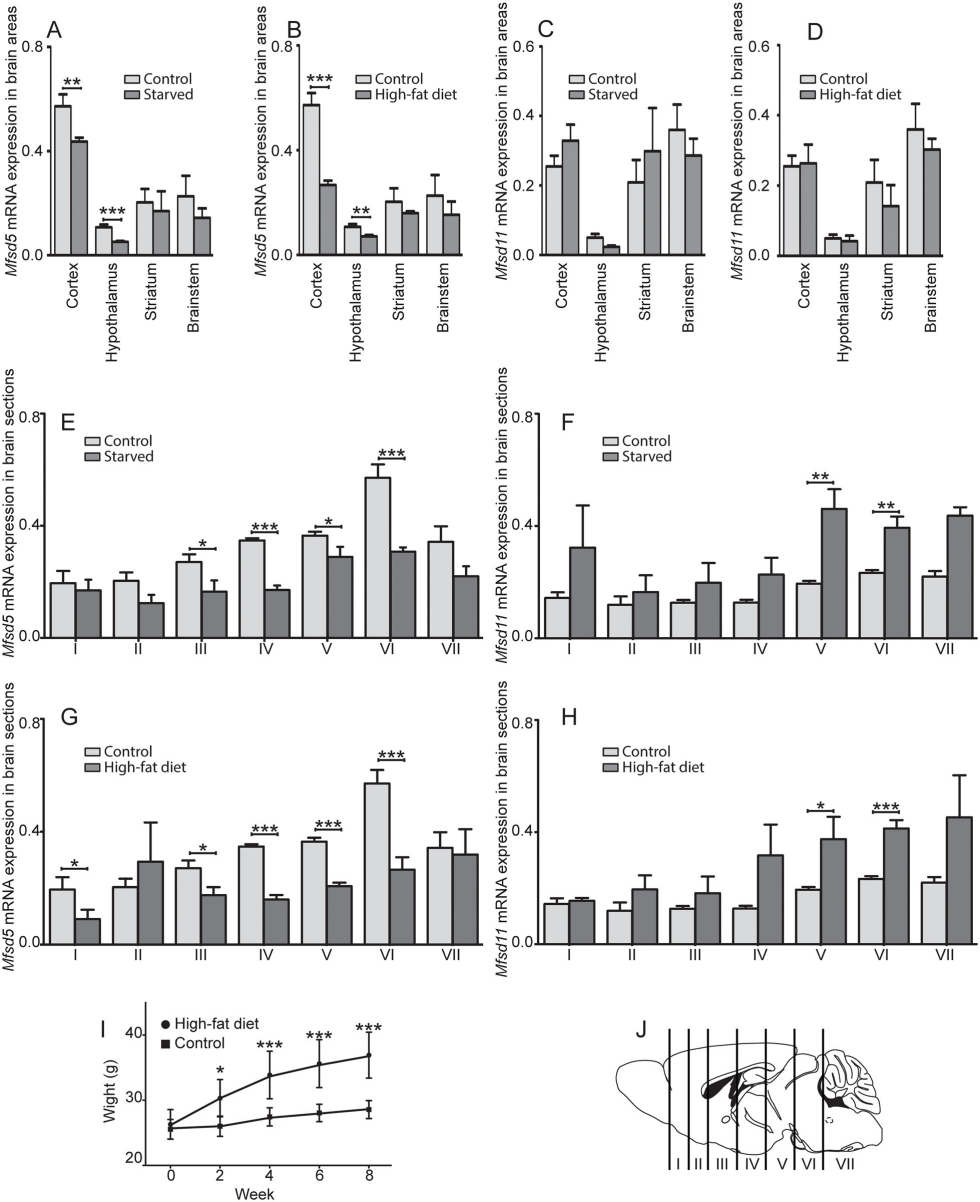
mRNA expression changes after starvation and HFD of *Mfsd5* and *Mfsd11*. The column charts display the normalized mRNA expression of *Mfsd5* and *Mfsd11*, with standard deviations (±SD). Mice were assigned different food; 1) normal chow (control), 2) food-deprived for 24h- and 3) high-fat diet. *Mfsd5* (A) and *Mfsd11* (B) expression in cortex, hypothalamus, striatum and brainstem samples from starved and obese mice. cDNA from four mice were pooled per sample. Effects of *Mfsd5* and *Mfsd11* expressions in sections from starved mice (n = 4) are depicted in C-D, while HFD (n = 6) samples are shown in E-F. Weight gain of mice in the HFD group compared to controls (I). A schematic description over how the seven brain cuts were made for section analyses (J). The schematic brain was adapted from Allen institute. *Gapdh*, *H3a* and *Actb* were used as housekeeping genes for all analyses. * corresponds to P≤ 0.05, ** to P≤ 0.01 and ***to P≤ 0.001.

We screened for expression changes in brain areas and sections from mice assigned to different food schemes; 1) fed normal chow (controls), 2) fed normal chow, but food-deprived for 24h before they were sacrificed and 3) fed HFD to induce obesity before qRT-PCR analyses. mRNA levels were measured specifically in cortex-, hypothalamus-, striatum- and brainstem samples where *Mfsd5* were reduced in cortex and hypothalamus after both starvation (P≤0.01) and HFD (P≤0.001), while no changes were seen in the striatum or brainstem samples (Fig 1A and 1B). For *Mfsd11*, no effects could be seen in any of the samples after either starvation or HFD (Fig 1C and 1D). When analysing the broad brain sections, *Mfsd5* was significantly down-regulated by starvation in brain section III (P≤ 0.05), IV (P≤ 0.001), V (P≤ 0.05) and VI (P≤ 0.001) (Fig 1E), while *Mfsd11* was up-regulated in section V (P≤ 0.01) and VI (P≤ 0.01) (Fig 1F). HFD altered the gene expression in a similar manner with reduced *Mfsd5* levels in brain section I (P≤ 0.05), III (P≤ 0.05), IV (P≤ 0.001), V (P≤ 0.001) and VI (P≤ 0.001) (Fig 1G), while *Mfsd11* was up-regulated in V (P≤ 0.05) and VI (P≤ 0.001) (Fig 1H) compared to the controls. The significant weight gain for the HFD mice is depicted in fig 1I, and for the specific sections, see fig 1J.

### The mRNA of *Mfsd5* and *Mfsd11* is abundantly expressed in various tissues in mice

**Fig 2:**
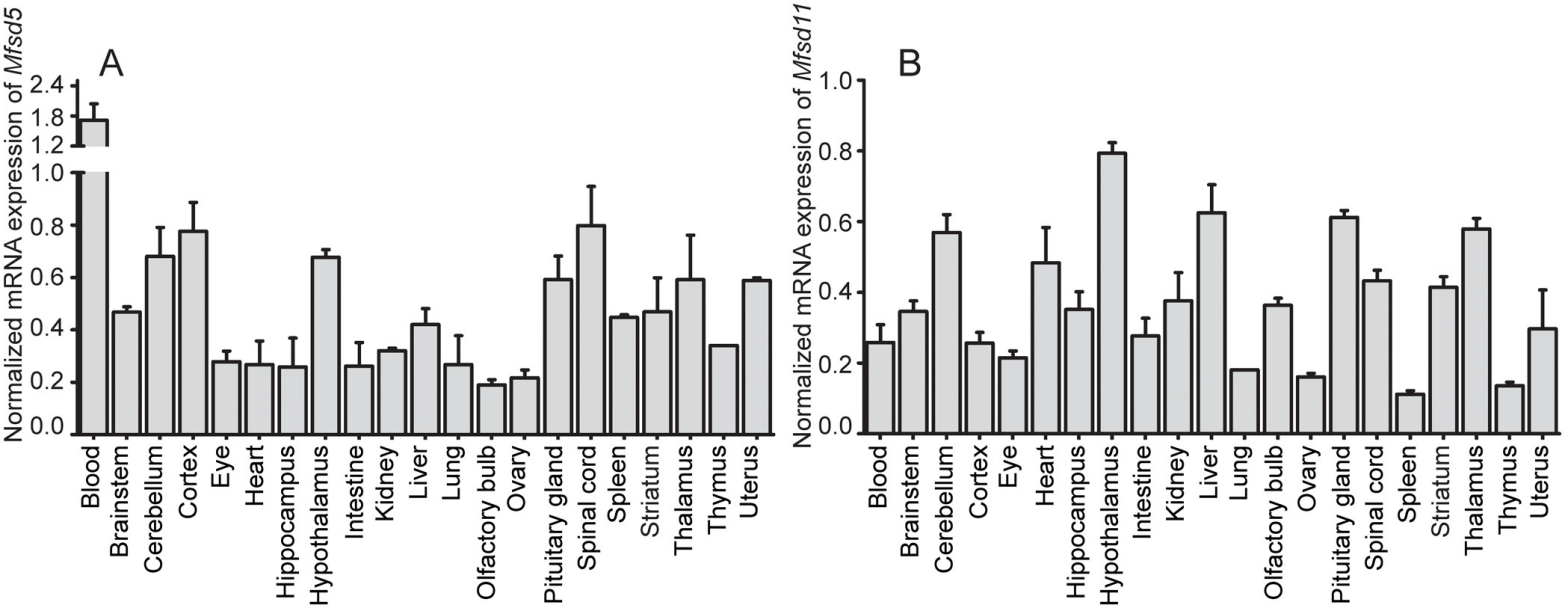
Normalized mRNA distribution. mRNA expression of *Mfsd5* and *Mfsd11* in wt mouse brain and peripheral tissues. cDNA from five mice were pooled for each organ. Samples were normalized against the geometric mean between the following reference genes: *Gapdh*, *bTub*, *Rpl19*, *Cyclo* and *Actb*. Plots show *Mfsd5* (A) and *Mfsd11* (B) expression, with standard deviations (±SD).

The normalized mRNA expression (±SD) of *Mfsd5* and *Mfsd11* in mice was explored by qRT-PCR. Specific central- and peripheral regions were isolated and used for qRT-PCR reactions. Both *Mfsd5* and *Mfsd11* showed abundant mRNA expression throughout the CNS as well as the periphery of the mouse body. The mRNA levels of *Mfsd5* varied throughout the panel; highest expression was observed in the blood (1.71±0.33 (normalized expression ± SD)) followed by CNS tissues such as cortex (0.77±0.11), hypothalamus (0.68±0.03), cerebellum (0.68±0.11) and spinal cord (0.80±0.15) (Fig 2A). The expression of *Mfsd5* in peripheral organs was generally lower than in brain, though it was still expressed at detectable levels in all tissues. Also, *Mfsd11* had high expression in specific brain areas; where the hypothalamus (0.79±0.03), thalamus (0.58±0.03) and brainstem (0.35±0.03) had among the highest expression (Fig 2B). However, there were peripheral organs with similar expression levels as the brain, where the liver (0.63±0.08) and heart (0.48±0.1) are two examples (Fig 2B). Hence *Mfsd11* was ubiquitously distributed throughout the mRNA panel with less divergence between central and peripheral regions.

### MFSD5 and MFSD11 are highly evolutionary conserved

**Fig 3:**
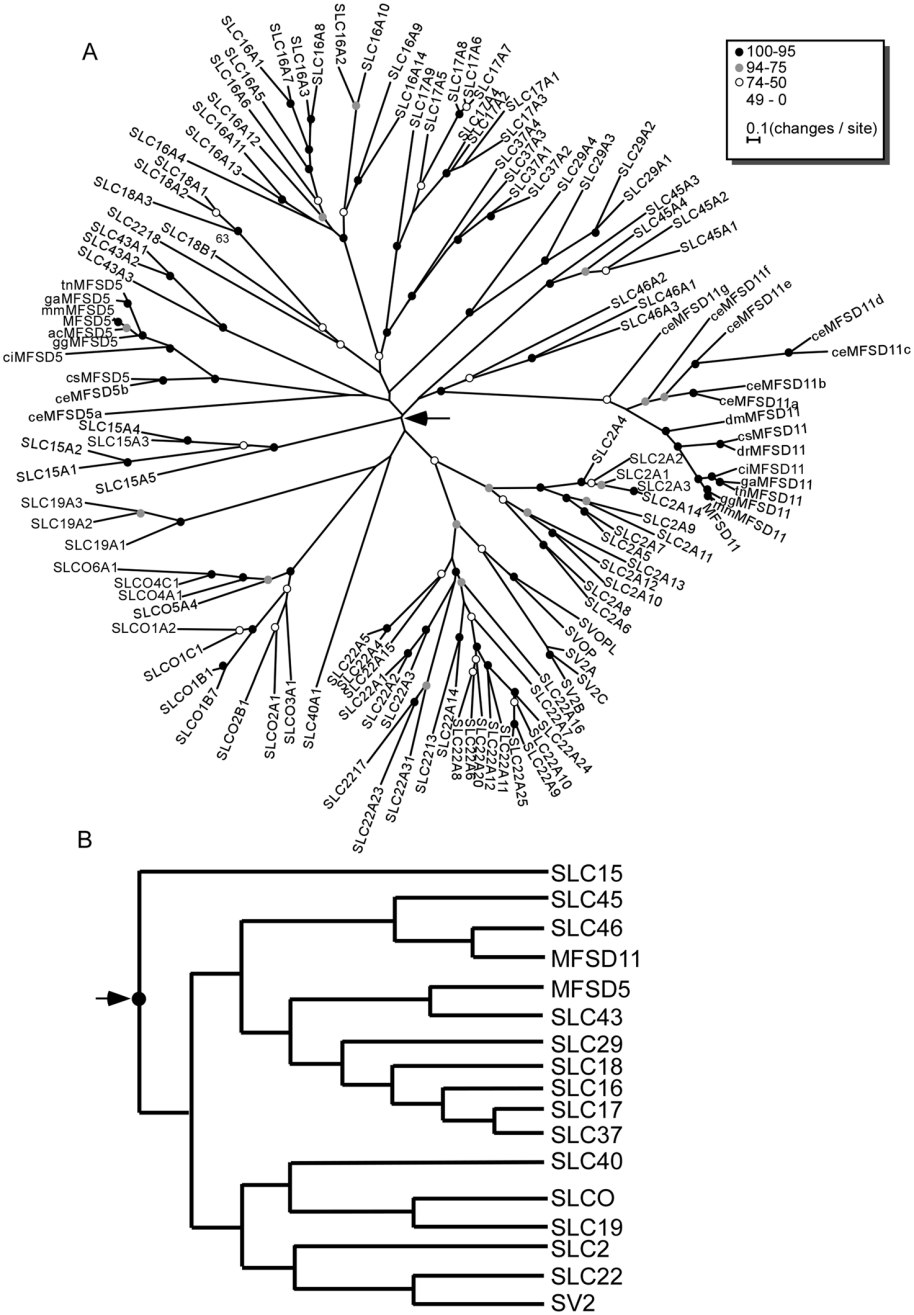
Phylogenetic analysis of the human SLCs from the MFS superfamily. (**A**) The phylogenetic tree was calculated using RAxML [24] with 500 bootstrap replicas. Circles on the nodes indicates the bootstrap support with black as 100% - 95%, grey 94% - 75% and white 74% - 50%. Nodes without circles had less than 50% bootstrap support. Species abbreviations: ac, *A*. *carolinensis*; ce, *C*. *elegans*; ci, *C*. *intestinalis*; cs, *C*. *savignyi*; dm, *D*. *melanogaster*; dr, *D*. *rerio*; gg, *G*. *gallus*; ga, *G*. *aculeatus*; no prefix, *H*. *sapiens*; mm, *M*. *musculus*; tn, *T*. *nigviridis*. (B) Schematic representation of the branching order obtained in the phylogenetic analysis.

We screened a set of 10 animal genomes, according to Table 2, along with the human genome to identify human orthologous of MFSD5 and MFSD11 and inferred the phylogenetic relationship of these protein sequences (Fig 3A). In this figure, nodes with 95% or higher bootstrap support are marked with a black dot; nodes with support between 94% and 75% are marked in grey while nodes with support between 74% and 50% are marked in white. Nodes with less than 49% supported are un-marked. We found MFSD5 and MFSD11 to be present in most species investigated, including the round worm *Caenorhabditis elegans* (*C*. *elegans*), suggesting that these two transporters are evolutionary old. Interestingly, we never identified more than one copy of neither MFSD5 nor MFSD11 in any specie except C. *elegans*. Also, we did not find MFSD5 in zebrafish or fruit fly (Fig 3A). Analysis of the branching orders within the MFS tree suggested that they clustered with SLC families having organic compounds as substrate profiles; where MFSD5 is most closely evolutionary related to the SLC43 family, while MFSD11 is most closely related to SLC46 (Fig 3B).

**Table 2:** Annotation of MFSD5 and MFSD11 sequences identified.

Species	MFSD5		MFSD11	
	Annotated name	Original sequence accession	Annotated name	Original sequence accession
*A*. *carolinensis*	acMFSD5	ENSACAP00000006600	---	---
*C*. *elegans*	ceMFSD5a	Y54G2A.45	scMFSD11a	M153.2
	ceMFSD5b	Y54G2A.4	scMFSD11b	F36G9.3
			scMFSD11c	F31D5.1
			scMFSD11d	Y52E8A.4
			scMFSD11e	F31D5.2.1
			scMFSD11f	C08D8.1
			scMFSD11g	C27C12.4
*C*. *intestinalis*	ciMFSD5	ENSCINP00000004183	ciMFSD11	ENSCINP00000013214
*C*. *savignyi*	csMFSD5	ENSCSAVP00000017916	csMFSD11	ENSCSAVP00000005637
*D*. *rerio*	---	---	drMFSD11	ENSDARP00000060452
*D*. *melanogaster*	---	---	dmMFSD11	FBpp0082101
*G*. *gallus*	ggMFSD5	ENSGALP00000023403	ggMFSD11	ENSGALP00000002730
*G*. *aculeatus*	gaMFSD5	ENSGACP00000012329	gaMFSD11	ENSGACP00000019646
*M*. *musculus*	mmMFSD5	ENSMUSP00000061997	mmMFSD11	ENSMUSP00000101971
*T*. *nigroviridis*	tnMFSD5	ENSTNIP00000018104	tnMFSD11	ENSTNIP00000014246

### Verification of antibodies specificities using western blot

**Fig 4:**
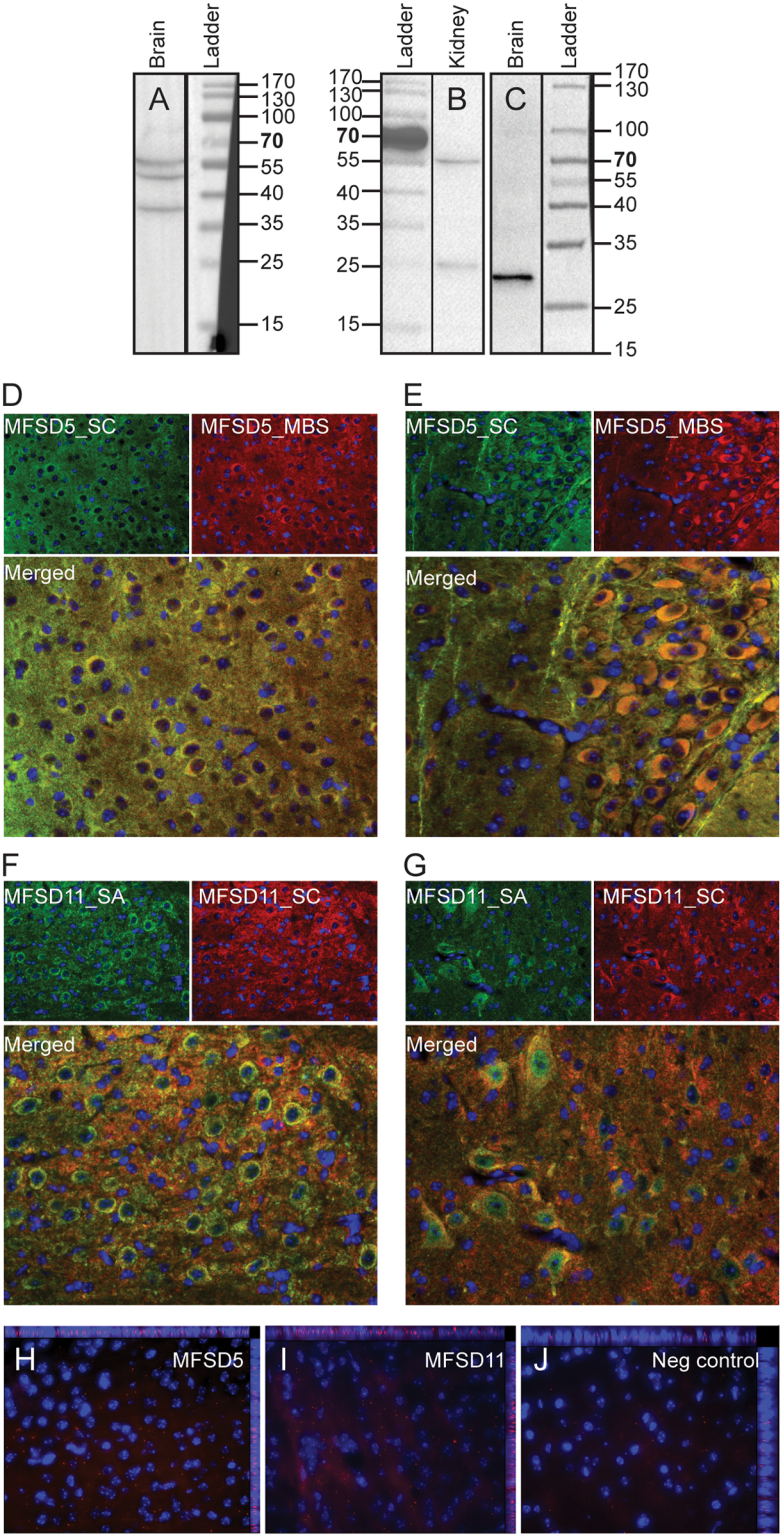
Antibody specificity. Western Blot was performed on fractionated wt mouse brain and kidney samples to verify antibody specificity. (A) The MFSD5 antibody (Santa Cruz) provided three bands in the brain fraction which correspond to the size of three splice variants of the proteins and the MFSD11 antibody(Sigma-Aldrich) (B) bound to two splice variants in the kidney sample and one in the brain fraction (C). Double immunohistochemistry showed overlap between antibodies from different vendors to verify specificity; MFSD5_SC (Santa Cruz) overlapped with MFSD_MBS (MyBioSource) (D, E) while MFSD11_SA (Sigma-Aldrich) co-stained with MFSD_SC (Santa Cruz) (F, G). The antibodies utilized throughout this manuscript are marked in green. Proximity ligation assay also showed interaction between the two MFSD5 antibodies (H) and the two MFSD11 antibodies (I), while no signal was detected in the negative control (J).

Western blot analyses were used to verify the specificity of the commercially available antibodies as were used for protein localization. Fractionated wt mouse brain and kidney were used for the western blot where the analysis showed three bands for anti-MFSD5 (Santa Cruz) at approximately at 40, 50 and 60 kDa in the brain fraction (Fig 4A), which matches the theoretical protein sizes of the three human splice variants: 45.3kDa [ensembl no: ENST00000551660], 49.8kDa [Accession no: NM_032889] and 61.5kDa [Accession no: NM_001170790]. The Sigma-Aldrich antibody for MFSD11 bound proteins at roughly 25 and 55kDa in the kidney sample (Fig 4B) and to proteins at 29kDA in the brain sample (Fig 4C) which correspond to the splice variants at size 22.9kDa [ensemble no: ENST00000585865] and the annotated mouse splice variant at 49.1kDa [Accession no: NM_178620]. Since both MFSD5 and MFSD11 are evolutionary conserved, it is highly possible that similar splice variants exist in both mice and humans. The fact that the sizes for MFSD11 are slightly larger than predicted are presumably due to posttranslational modifications as the protein sequence contains three strong N-glycosylation sites, as predicted by the NetNGlyc server [13].

### Verification of antibodies specificities in tissues

Since the succeeding data relies on antibodies, we verified the staining in tissue as well by labelling the MFSD5 and MFSD11 proteins with antibodies from different vendors. The MFSD5 antibody from Santa Cruz bind an epitope at the N-terminal and it was co-stained with a MFSD5 marker from MyBioSource (C-terminal epitope), and these antibodies overlapped completely, as can be seen in the merged images depicted in Fig 4D and 4E. The MFSD11 antibody from Sigma-Aldrich (internal epitope) co-localized with the MFSD11antibody from Santa Cruz (internal epitope) (Fig 4F and 4G). To adequately evaluate the antibodies used, we run PLA using the two antibodies against the same protein. The fluorescent signal seen in Fig 4H (MFSD5) and Fig 4I (MFSD11) implies that the two antibodies from different companies bound within 40nm to each other, which suggests that they bound the same protein. Hence, that two antibodies from different vendors label the same protein strengthens our confidence in the specificity of the antibodies.

### Abundant expression of the MFSD5 and MFSD11 proteins in the mouse brain

**Fig 5:**
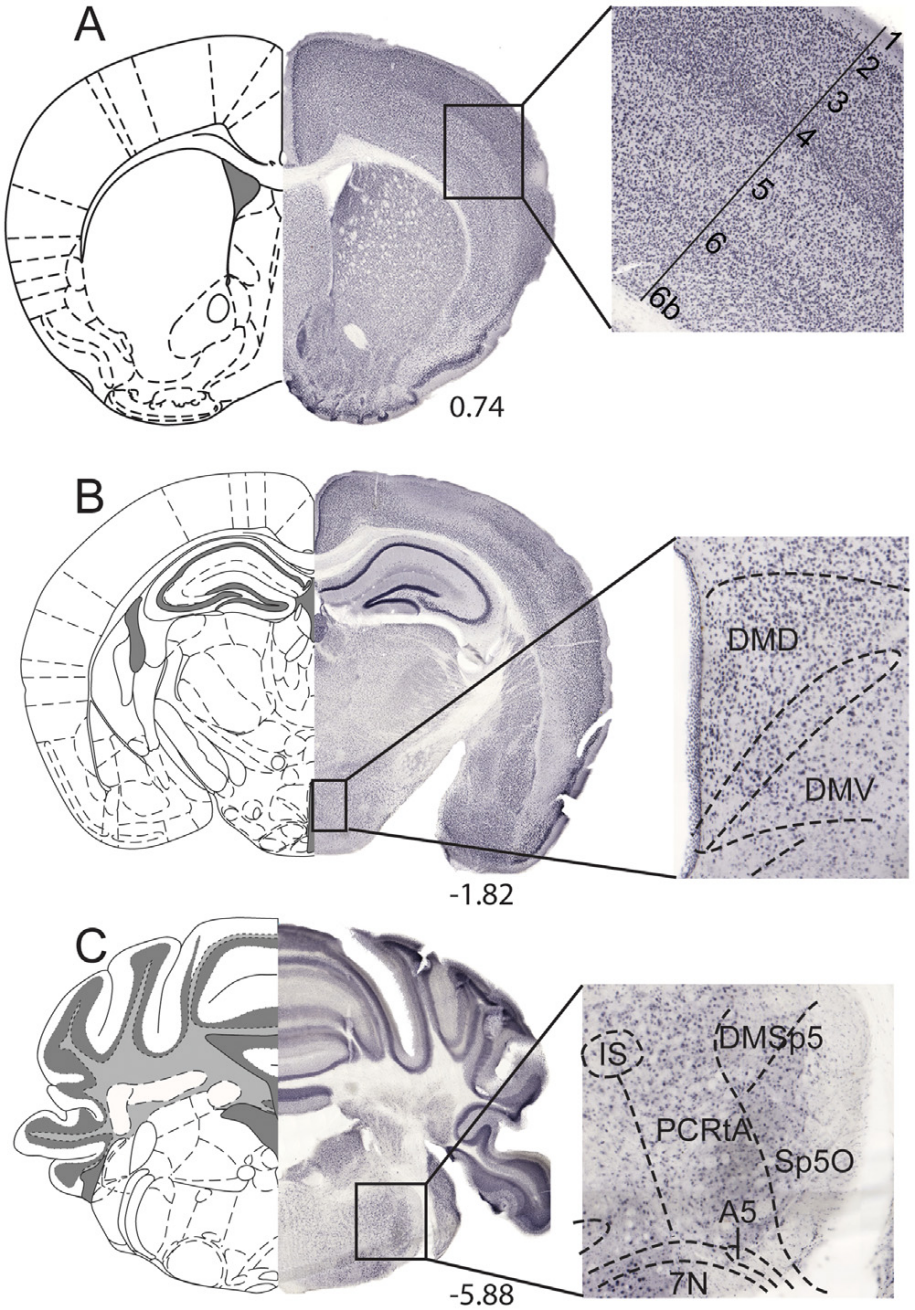
Abundant MFSD11 protein expression. DAB-immunohistochemistry stained MFSD11 on 70μm wt floating brain sections. Overview images with specific regions magnified. (A) Staining pattern in cortex, (B) hypothalamic nuclei and the (**C**) brainstem. The schematic bregma regions were modified from *The mouse brain* [28].

The protein expression pattern of MFSD11 was examined in 70μm coronal sections of mouse brain through immunohistochemistry with DAB staining, using an anti-MFSD11 antibody. This showed similar expression as the ubiquitous mRNA expression, with abundant MFSD11 protein staining throughout the brain. When studying the global MFSD11 protein expression, high staining was for example observed in the cortical layers (magnified in Fig 5A) hypothalamus (magnified in Fig 5B) 4E and brainstem (magnified in Fig 5C). Protein expression for MFSD5 was investigated with fluorescent staining on paraffin sections due to insufficient detection of MFSD5 expression using DAB staining (data not showed). MFSD5 has a broad staining throughout the brain with high labelling in the cortex, hypothalamus and cerebellum. Hence, the MFSD5 and MFSD11 protein expression pattern overlapped well with the mRNA distribution.

### MFSD5 and MFSD11 are exclusively expressed in neurons in the mouse brain

**Fig 6:**
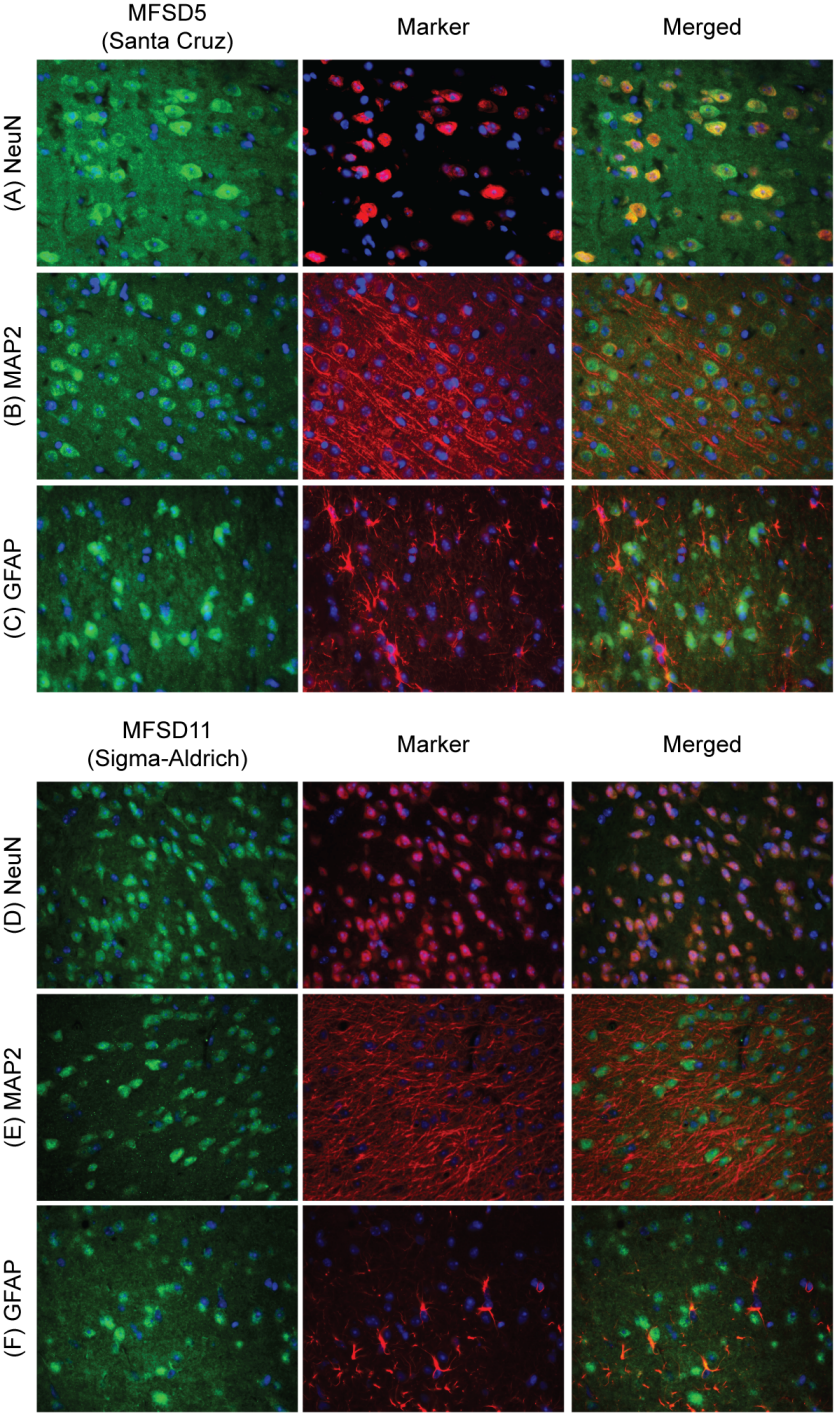
Neuronal expression of MFSD5 and MFSD11 in the mouse brain. Double immunohistochemistry with fluorescent markers stained on 7μm wt mouse coronal paraffin sections. MFSD5 and MFSD11 were labelled in green, markers (NeuN, MAP2 and GFAP) in red and cell nuclei (DAPI) in blue. MFSD5 co-localized with the neural marker NeuN (A), but not with the neural dendritic marker MAP2 (B) or the astrocytic glial cell marker, GFAP (C). Also the MFSD11 antibody co-localized with NeuN (D), but not with MAP2 (E) or GFAP (F).

Double immunohistochemistry with fluorescent markers was performed on paraffin sections to determine if the genes of interest were expressed in neurons or glial cells. A high degree of co-localization of anti-MFSD5 and anti-MFSD11 antibody with the neuronal marker NeuN [29] was observed throughout the brain (Fig 6A and 6D), where both MFSD5 and MFSD11 were located to the soma of neurons. Neither MFSD5 nor MFSD11 co-localized with the neuronal microtubule-associated protein 2 marker, MAP2 [30] (Fig 6B and 6E), or with the astrocyte maker glial fibrillary acidic protein, GFAP [31] (Fig 6C and 6F). This suggests that MFSD5 and MFSD11 were exclusively expressed in the neuronal soma in the mouse brain.

### MFSD5 and MFSD11 are abundantly expressed in e14-15 embryos

**Fig 7:**
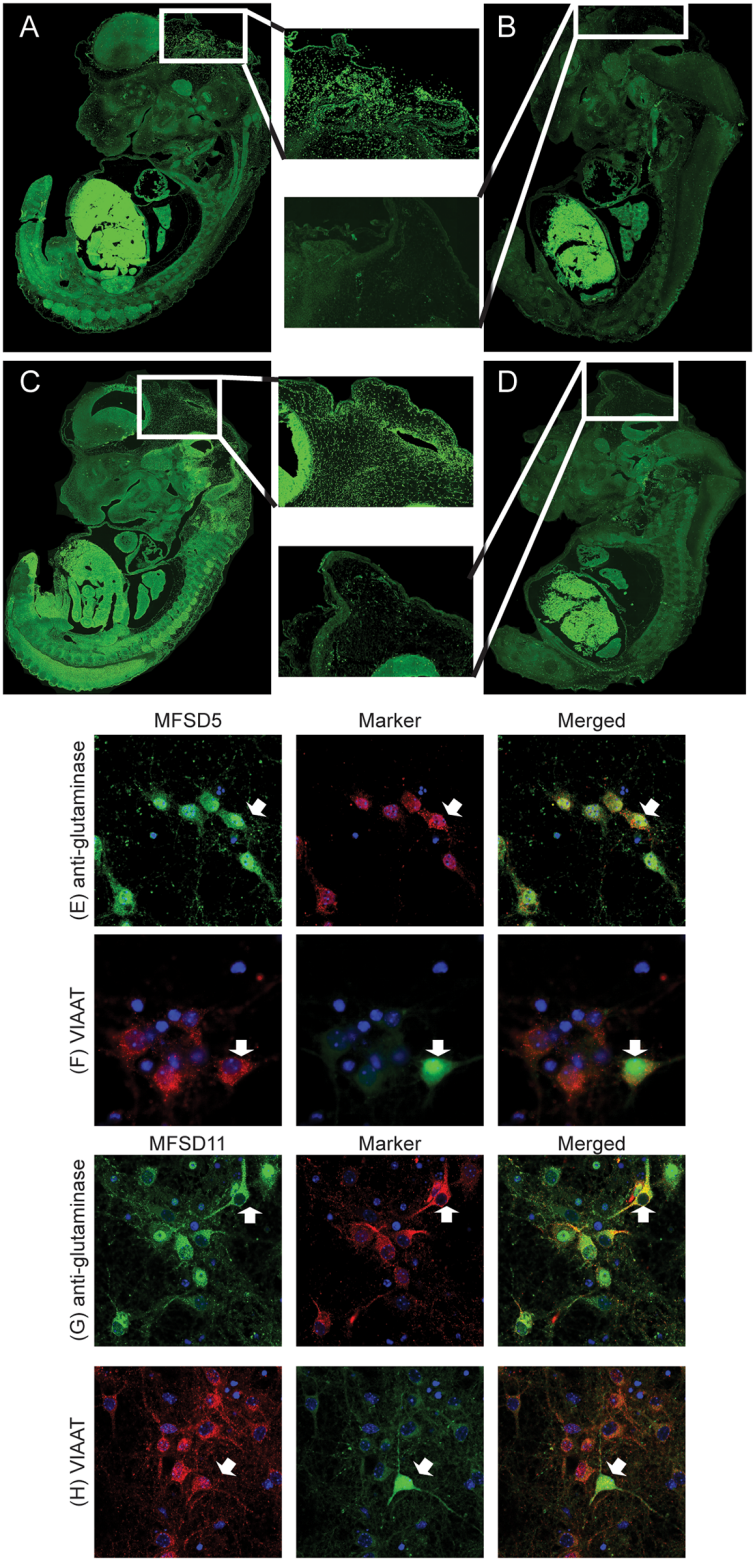
Embryonic and neuronal expression of MFSD5 and MFSD11. MFSD5 antibody labelling in mouse e14-15 embryos are shown in A, with subsequent negative control (B). C depicts the MFSD11 staining in embryo, where D is its negative control. (E) MFSD5 in green and glutamate marker anti-glutaminase in red, co-localized, exemplified in cell indicated by the white arrow. (F) MFSD5 in red and eGFP-marked inhibitory vesicles, VIAAT, in green showed co-localization, depicted by white arrow. (G) MFSD11 in green and glutamate marker anti-glutaminase in red, co-localized, exemplified in cell indicated by the white arrow (H) MFSD11 in red and eGFP-marked inhibitory vesicles, VIAAT, in green showed co-staining, depicted by white arrow. Cell nucleus staining DAPI in blue was included in all staining.

To visualize protein expression during embryonic development, immunohistochemistry was performed on mouse embryo sagittal sections from embryonic day 14–15. MFSD5 staining displayed high protein expression in the midbrain (Fig 7A) and scattered expression elsewhere. MFSD11, on the other hand demonstrated abundant staining with distinctive labelling in the vertebrates and the brain (Fig 7C). The staining observed in liver was considered unspecific since both negative controls (Fig 7B and 7D), stained only with secondary antibody, also displayed high antibody binding there.

### Expression in excitatory and inhibitory neurons

Wt primary cortex cultures were used to co-stain anti-MFSD5 and anti-MFSD11 with the glutamate marker anti-glutaminase, to analyse if the transporters were expressed in excitatory neurons. MFSD5 and MFSD11 staining on primary cortex cells from VIAAT-eGFP embryos were subsequently used to investigate expression in inhibitory neurons.Both MFSD5 and MFSD11 co-localized with the glutamate marker anti-glutaminase (Fig 7E and 7G), and with the VIAAT-eGFP positive cells (Fig 7F and 7H). Hence, MFSD5 and MFSD11 were expressed in both excitatory and inhibitory neurons.

## Discussion

The closely related major facilitator superfamily proteins MFSD5 and MFSD11 have never been studied in detail, even though possible functions for MFSD5 has been discussed previously [14,17], and that the expression of *Mfsd11* is known to change after amino acid starvation in immortalized cell cultures [18]. Here we show that both *Mfsd5* and *Mfsd11* are regulated by dietary status, in specific mouse brain areas and broader brain sections, suggesting a potential involvement in the energy homeostasis. This is further strengthened since MFSD5 has been reported to interact with GLP-1R [17], which is an important regulator of the energy balance.

We employed a starvation assay to study if the expressions changed in mice exposed to certain food paradigms. Mice were food deprived for 24h or fed HFD before qRT-PCR analyses. Both brain specific areas and brain sections were studied. *Mfsd5* was specifically down-regulated after both starvation and HFD in cortex and hypothalamus, while no changes were measured in striatum or brainstem. Furthermore, significant down-regulation of *Mfsd5* was detected by both starvation and HFD in brain sections including physiological areas integrating the reward circuits and feeding regulation. For example, in section III there was a down-regulation of about 39% by starvation and 35% by HFD, while in section IV there was a significant reduction of 51% after starvation and 54% by HFD compared to the normal fed controls. These sections (III and IV) for example include the hypothalamic nuclei, a well-known component in the food intake regulation [32] and the amygdala, which contains important nuclei for feeding regulation, reward and anxiety [25,26,33,34]. Down-regulation was also seen in section V (21% starvation, 43% HFD) and in section VI (46% starvation, 53% HFD).

Since MFSD5 and MFSD11 are phylogenetically closely related, it was interesting to find that *Mfsd11* responded with up-regulation to energy homeostasis alterations, hence in the opposite manner to *Mfsd5*. *Mfsd11* expression is up-regulated following amino acid deprivation in hypothalamic cells [18] and we showed that it was also attenuated in mouse brain sections following food deprivation or HFD. Though, no changes were detected when studying certain brain areas. While looking at the expression in the brain sections, *Mfsd11* mRNA levels were significantly up-regulated in section V and section VI with 138% and 69% respectively following starvation, while HFD resulted in increased expression of 93% and 77% in the same brain sections compared with controls. Section V contain for example the substantia nigra pars compacta, one of several locations for the midbrain dopaminergic neurons [35], while section VI include the ventral tegmental area which enclose dopamiergic cells projecting to the striatum and nucleus accumbens [35,36], making it one of the most basic component in the brain reward circuit.

The benefit with studying brain sections is that the whole brain is included. Since neurons project throughout the brain, this is a way to include whole circuits. This is also a good way to study overall expression changes in the brain, and it narrows down possible areas responsible for the detected changes. However, it can be considered speculative since each section include several areas with different functions. Therefore we included some specific brain areas as well, where the reduction of *Mfsd5* in specific brain areas corresponded with the effects seen in the brain sections, supporting the hypothesis that it is involved in food- and reward circuits. Based on the brain sections we also suggests that *Mfsd11* is affected by energy intake, but that these effects are due to other brain areas than cortex, hypothalamus, striatum and brainstem.

The C57BL/6J mouse strain we use is a commonly used model when studying the metabolic syndrome [37] since it respond in similar way as humans, with both inflammation and a stress response as secondary effects. The changed gene expressions of *Mfsd5* and *Mfsd11* could hence be due to secondary effects in excess of energy regulation. The high blood levels of *Mfsd5* propose an involvement in the immune system, making it plausible to consider the reduced expression as an inflammatory response. However, we find it more likely that *Mfsd5* is primarily involved in energy homeostasis since it was not associated with the immune response when searched for it in various databases [17]. SLCs involved in the immune response also seem to be more often up-regulated during inflammation [38–40], whereas *Mfsd5* was down-regulated.

Noteworthy is that both genes responded opposite each other, but similar towards both food alterations; where *Mfsd5* was down-regulated and *Mfsd11* was up-regulated. This has previously been seen for SLCs with organic substrate profiles, and it can be explained by several molecular machineries [41] that are involved in keeping the energy homeostasis. As an example, there are two major amino acid sensing pathways; the general control non-depressible (GCN) pathway, which is also called the amino acid responsive (AAR) pathway, and the mammalian/mechanistic target of rapamycin complex 1 (mTORC1) [41,42]. Mammalian cells can regulate the protein synthesis based on these pathways [42]. The AAR pathway is activated during amino acid starvation and controls the amino acid concentration that is required for adequate protein synthesis [43]. After the AAR activation, the cell increases transcription of genes containing AAREs (amino acid responsive elements) [43], and there are SLC amino acid transporters with characterized AAREs, where SLC38A2 (SNAT2) [44,45] is an example, meaning that it can control the expression of SLC transporters. When, for example, hypothalamic cells were deprived of all amino acids, several SLCs along with MFSD11 were upregulated [18].

mTORC1 is, on the other hand, activated when the cells have sufficient levels of amino acids [46] to ensure protein synthesis and cellular growth, and this pathway could be activated during HFD in mice. Activation of the mTORC1 pathway indirectly increases protein synthesis, since it, after a kinase cascade, phosphorylates the S6 kinase that increases the translation of mRNA species coding for ribosomal proteins [42,47]. This in turn will keep the protein synthesis and cell growth at a rate consistent with nutrient availability [47]. Thus, the two pathways alter the protein synthesis during opposite circumstances [42]. SLC38A2 is suggested to be an activator of the mTOR pathway [48,49], showing that SLC members has been identified to be involved in both pathways [44,48,49]. Hence the up-regulation of *Mfsd11* after starvation could be a result of AAR activation, while the HFD could activate *Mfsd11* expression via the mTORC1 pathway.

Taken everything together, that *Mfsd5* and *Mfsd11* expression are affected by changes in food intake and that they phylogenetically cluster with SLC families with organic substrate profiles, we find it highly possible that they have organic substrate profiles and that they are involved in energy homeostasis.

Based on the phylogenetic analysis performed here, we suggest that MFSD5 and MFSD11 could be classified as SLCs in humans, and we show that they are evolutionary highly conserved. Both genes clustered with SLC families having organic substrate profiles, and since SLC families with similar substrate profiles usually cluster together [50], we find it possible that they transport organic molecules as well. Though MFSD5 has previously been suggested to transport inorganic ions [14]. Some of the closest related families are sugar transporters (SLC45, SLC37 and SLC22) while others have amino acids (SLC43 and SLC17) or mono-amines (SLC18) as substrates.

It was previously shown by qRT-PCR in rats [8] and by *in situ* hybridisation in mouse embryos [13] that *Mfsd5* is widely expressed, while the location of *Mfsd11* was unknown until our study was performed. We used qRT-PCR to extend the expression study of *Mfsd5* and *Mfsd11* to both central and peripheral mouse tissue, where both genes showed abundant expression throughout the mouse. *Mfsd5* showed a tendency towards higher expression in brain areas, while *Mfsd11* displayed less difference between central and peripheral regions. This corresponds well with previous qRT-PCR studies of *Mfsd5* performed on rat tissue; where a similar trend towards higher expression in brain regions can be seen [8].

The protein localization of MFSD5 and MFSD11 has until now been unknown, but here we reveal the first histology map obtained by immunohistochemistry staining using commercially available antibodies. We have explored where and in which specific cells in the mouse brain MFSD5 and MFSD11 were expressed, as well as their location in mouse embryos to verify their early on-set, which enhances their importance also during development. Finally, we determined by immunocytochemistry that MFSD5 and MFSD11 are expressed in glutamatergic and GABAergic neurons in embryonic primary cell cultures.

The antibodies used to study MFSD5 and MFSD11 were verified in three ways: 1) western blot, 2) co-staining with antibodies from different vendors and 3) PLA with antibodies directed against different epitopes. Western blot analysis on fractionated mouse brain revealed three bands for MFSD5, which corresponded with the predicted protein size of the three human splice variants, and since there is 96% similarity between the human and mouse protein sequence [8], it is reasonable that the same splice variants exists in both species. MFSD11 bound proteins of two different sizes in the fractionated kidney sample and they corresponded well with human and mouse splice variants. In the mouse brain fraction, only the smaller MFSD11 splice variant, annotated in humans, was detected after extensive protein denaturation. That the protein sizes were slightly heavier than predicted for MFSD11 could depend on posttranslational modifications, which is highly possible since MFSD11 contain three strong N-glycosylation sites. This could also be the reason why the lighter kidney band and the band found in the brain had slightly different sizes; that the protein is glycosylated differently in the brain verses the periphery. One other possible explanation is that the brain sample required harder denaturation to be detectable, and that this influenced the protein migration through the gel. To ensure that the antibodies were specific also in tissues, double immunohistochemistry were performed using antibodies from different vendors. Anti-MFSD5 (Santa Cruz) was co-labelled with anti-MFSD5 (MyBioSource), while anti-MFSD11 (Sigma-Aldrich) was stained with anti-MFSD11 (Santa Cruz). This showed complete overlap between the antibodies, indicating that they bound the same protein. By PLA we also visualise the interaction between the antibodies, meaning that they bound within proximity to each other. Taken everything together, the western blot, fluorescent co-staining and PLA, it verifies the antibody specificity.

Bright field staining with an anti-MFSD11 and fluorescent staining with and anti-MFSD5 antibody visualized a high abundance in the mouse brain including ubiquitous staining in for example cortex, hypothalamus and brainstem, regions where we also measured mRNA expression changes after altered food intake. Thus, MFSD5 and MFSD11 displayed similar mRNA and protein patterns. Furthermore, double-staining with the neural marker NeuN [29] revealed staining of MFSD5 and MFSD11 in neurons, while no co-localization was observed with neither the neuronal dendritic marker MAP2 [30] nor with the astrocytic cells marker GFAP [31]. The staining’s were located around the soma, but not in the nuclei, as seen when comparing the protein staining and PLA signal with DAPI. By staining sagittal sectioned e14-15 embryos, MFSD5 displayed extensive midbrain signals and a scattered body expression was visualized, while MFSD11 had an abundant expression pattern in both the brain and the periphery. The liver displayed high fluorescence levels in the MFSD5 and MFSD11 images; however, since the negative controls also showed similar labelling of the liver we consider this unspecific.

Double-staining also showed that MFSD5 and MFSD11 are staining in accordance with the excitatory neural marker anti-glutaminase [51,52] and with the inhibitory neuronal VIAAT-eGFP labelling in primary culture. Taken together, MFSD5 and MFSD11 were expressed in excitatory and inhibitory neurons, throughout development and in adult mice.

## Conclusions

We have performed a detailed histological analysis of the membrane bound transporters MFSD5 and MFSD11 as well as the first functional study where we examined their presumed involvement in food intake. We showed that MFSD5 and MFSD11 belong to the SLC family in humans and that they are highly evolutionary conserved. It is also plausible to consider organic substrates for both transporters due to their relatedness to SLCs with known organic substrates. Furthermore, we showed recurrent expression changes when exposed to different food paradigms, starvation or HFD, in mouse tissues, which could also be explained if they have an organic substrate profile. Their mRNA and protein distribution in the adult mouse brain showed abundant expression. And that both proteins were expressed during embryogenesis suggests they are involved in vital functions. Fluorescent immunohistochemistry further revealed that MFSD5 and MFSD11 were expressed in both excitatory and inhibitory neurons.
